# Prevalence, health burden, and occupational risk factors of work-related musculoskeletal disorders among informal automobile repair artisans in Sunyani Municipality, Ghana: a cross-sectional study

**DOI:** 10.1186/s12889-026-28478-8

**Published:** 2026-07-08

**Authors:** Bosiako Yaw Antwi, Eric Yiadom Boakye, Jacob Solomon Idan, Martin Muonibe Beru, Eugene Sackeya, John Nuurpo, Aliyu Mohammed

**Affiliations:** 1 Department of Occupational and Environmental Health and Safety, School of Public Health, KNUST, Kumasi, Ghana; 2 Department of Health Policy, Management and Economics, School of Public Health, KNUST, Kumasi, Ghana; 3 Department of Epidemiology and Biostatistics, School of Public Health, KNUST, Kumasi, Ghana; 4 Department of Surgery, Tamale Teaching Hospital, Tamale, Ghana

**Keywords:** Musculoskeletal disorders, Informal sector, Auto mechanics, Ergonomics, DALYs, Occupational health, Ghana

## Abstract

**Background:**

Work-related musculoskeletal disorders are one of the major causes of disability globally. There is a lack of evidence in the Ghanaian context, especially in smaller urban areas and various auto-related trades carried out in the same clusters of informal workshops. This study evaluated the prevalence, health burden, and occupational risk factors of work-related musculoskeletal disorders among automobile repair artisans in the Sunyani Municipality of Ghana.

**Method:**

A cross-sectional study was carried out among 271 informal auto repair artisans in the Sunyani Municipality between November and December 2025. Data were collected using a structured questionnaire that included the Standardized Nordic Musculoskeletal Questionnaire, work-related ergonomic exposure questions, and quality-of-life questions. Factors associated with the prevalence of work-related musculoskeletal disorders in 12 months were determined using multivariable logistic regression. Disability-adjusted life years were calculated using the Global Burden of Disease method. Analysis was done using Stata version 17. Significance was set at *p* < 0.05.

**Results:**

The study involved 271 participants, predominantly male (95.9%), with a median age of 33 years. Most were young adults aged 18–29 (40.6%). The prevalence of work-related musculoskeletal disorders was 52.1%. The most affected body parts were the lower back (42.8%), shoulders (32.5%), and upper back (26.6%). Frequent bending/twisting (94.8%), standing for prolonged periods (83.4%), repetitive hand/wrist movements (73.4%), and the use of vibrating tools (66.8%) were common risk factors. In adjusted analyses, having height 1.67–1.81 m (aOR = 3.42, 95% CI: 1.43–8.18), monthly income GHS 1501–2000 (aOR = 4.61, 95% CI: 1.69–12.58), use of vibrating tools (aOR = 0.56, 95% CI: 0.31–0.99), and job dissatisfaction (aOR = 0.19, 95% CI: 0.05–0.66) were independently associated with work-related musculoskeletal disorders. Total disability-adjusted life years lost were 62.21, with mechanics and welders contributing the highest shares.

**Conclusion:**

Work-related musculoskeletal disorders impose a substantial health and productivity burden on automobile repair artisans. Modifiable ergonomic and organizational factors to compensate for the height and increased number of daily tasks are key to preventing injuries and focusing on health surveillance among automobile repair artisans in Ghana.

**Supplementary Information:**

The online version contains supplementary material available at https://doi.org/10.1186/s12889-026-28478-8.

## Background

Work-related musculoskeletal disorders (WMSDs) are among the leading contributors to disability and reduced work capacity globally, accounting for a substantial proportion of years lived with disability across diverse occupational groups [1, 2]. Work-related musculoskeletal disorders comprise a wide range of inflammatory and degenerative conditions affecting muscles, tendons, joints, ligaments, and nerves, often resulting from prolonged exposure to physical, ergonomic, and psychosocial workplace hazards [3].

Globally, musculoskeletal disorders affect more than 1.7 billion people, with low back pain consistently ranked as the single largest cause of disability worldwide [4]. Evidence also indicates that the burden is disproportionately shouldered by workers in manual, low-skilled, and informal sectors because of the lack of regulatory protection, poor ergonomics, and limited access to occupational health services [5, 6]. In sub-Saharan Africa, the informal sector employs the majority of the working population and plays a critical role in urban economies; however, it remains largely neglected within occupational health research and policy frameworks [7]. In Ghana, informal employment accounts for over 80% of total national employment, with auto repair artisans constituting a prominent occupational group within urban and peri-urban settings [8]. Informal auto repair work is characterized by physically demanding tasks such as prolonged bending and twisting of the trunk, sustained standing, squatting or kneeling, repetitive hand and wrist movements, overhead work, manual handling of heavy vehicle components, and frequent use of vibrating tools. These exposures are well-established risk factors for the development of work-related musculoskeletal disorders [9, 10]. Previous studies conducted among auto mechanics and related trades in Ghana and other African countries have reported a high prevalence of work-related musculoskeletal disorders, particularly affecting the lower back, shoulders, neck, and upper limbs [9–11].

Nevertheless, much of the existing literature in Ghana and other low- and middle-income countries has focused on individual automobile-related occupations or specific anatomical regions, limiting understanding of the cumulative musculoskeletal burden across multiple automobile repair trades operating within shared informal workshop environments. In addition, few studies have quantified the disability burden associated with WMSDs among informal automobile repair artisans using disability-adjusted life years (DALYs). Given the limited occupational health surveillance and ergonomic interventions within Ghana’s informal automobile sector, there is a need to generate evidence on the prevalence, determinants, and health burden of WMSDs to inform targeted occupational health policies and preventive interventions. In this study, health burden refers to the disability burden associated with WMSDs, quantified using disability-adjusted life years (DALYs), particularly among informal sector workers [4, 5].

Additionally, evidence remains limited regarding the combined influence of socio-demographic characteristics, ergonomic exposures, and psychosocial factors on work-related musculoskeletal disorders among informal auto repair artisans in Ghana. However, filling these gaps is critical in creating context-specific evidence for targeted ergonomic actions, occupational health education, and policy measures to mitigate preventable disability in the informal sector [3]. Therefore, this study assessed the prevalence, anatomical distribution, determinants, and health burden of work related musculoskeletal disorders among informal automobile repair artisans in Sunyani Municipality, Ghana, and estimated the associated disability burden using disability adjusted life years.

## Materials and methods

### Study design and setting

A descriptive cross-sectional study design was used to assess the prevalence and determinants of work-related musculoskeletal disorders (WMSDs) among automobile repair artisans. The study was conducted at Sunyani Magazine, a prominent automobile repair hub in the Sunyani Municipality, Bono Region, Ghana. The municipality is a rapidly growing urban center spanning 829.3 km^2^ with a 2020 projected population of 147,982 [12]. Sunyani Magazine serves as a central cluster for various informal sector artisans, including mechanics, welders, electricians, vulcanizers, sprayers, and panel beaters. These workers are routinely exposed to significant occupational hazards, including repetitive movements, prolonged standing, awkward postures, and heavy lifting. This site was selected for its high concentration of automobile artisans, providing a representative setting to investigate work-related musculoskeletal disorders (WMSDs) in Ghana’s informal economy.

### Study population

The study population consisted of informal auto repair artisans working at the Sunyani Magazine. These workers were engaged in different automobile repair-related activities, including mechanical repairs, electrical repairs, welding, spray painting, vulcanizing, and panel beating. The population comprised workers who performed physically demanding tasks as part of their daily occupational responsibilities. The target population included all active informal auto repair artisans who had been working in the automobile repair industry within the study area and were available during the period of data collection. Participants were considered eligible for inclusion if they were actively engaged in automobile repair work, had worked in the occupation for at least one year, were aged 18 years or older, and provided informed consent to participate in the study. Individuals who were absent from different categories from the workplace during the data collection period, had a known history of musculoskeletal disorders unrelated to occupational exposure, or declined to participate were excluded from the study.

### Sample size determination

The sample size for the study was determined using Cochran’s formula for estimating a single population proportion:

$$\:n=\frac{{z}^{2}\times\:p(1-p)}{{d}^{2}}$$

The calculation was based on an expected prevalence of work-related musculoskeletal disorders obtained from previous studies conducted among similar occupational groups with *P* = 0.72 according to [13]. A confidence level of 95% (Z = 1.96) and a margin of error of 5% were used in the calculation. The computed minimum sample size was subsequently adjusted to account for potential non-response by adding a percentage to the calculated value. After this adjustment, the final sample size for the study was determined to be 271 participants, which was considered adequate to ensure reliable estimation of prevalence and sufficient statistical power to assess associations between occupational risk factors and work-related musculoskeletal disorders among informal auto repair artisans.

### Sampling method

A stratified random sampling technique was used to select participants for the study. A line-list of registered automobile repair artisans was obtained from the executive leadership of Sunyani Magazine and artisan association records. The artisans were subsequently grouped into strata according to occupational category, including mechanics, welders, electricians, vulcanizers, sprayers, and panel beaters. Proportional allocation was then applied to determine the number of participants selected from each occupational group to ensure fair representation of all categories of workers. Within each stratum, a simple random sampling technique using the lottery method was used to select participants. Eligible artisans in each stratum were assigned unique identification numbers, which were written on separate pieces of paper, thoroughly mixed, and randomly drawn without replacement until the required sample size for that stratum was obtained. This approach ensured that every worker who met the inclusion criteria had an equal chance of being selected for participation in the study and enhanced the representativeness and generalizability of the study findings.

### Data collection and management

Data were collected using interviewer-administered electronic questionnaires deployed through Kobo Collect. The questionnaire collected information on respondents’ socio-demographic characteristics, work history, ergonomic exposure, and musculoskeletal symptoms. The standardized Nordic Musculoskeletal Questionnaire (NMQ) was adapted to collect information on the 12-month prevalence of work-related musculoskeletal disorders for different anatomical regions, and it is widely used and validated instrument for assessing musculoskeletal symptoms as well as the quality of life and loss of productivity [14]. These variables were selected based on previous epidemiological and ergonomic studies that have identified socio-demographic, anthropometric, occupational, and psychosocial factors as potential determinants of work-related musculoskeletal disorders among manual workers and automobile repair artisans [10, 11–15]. In addition, the number of children was included as a proxy measure of family responsibility and socioeconomic burden, as workers with greater family obligations may undertake longer working hours, heavier workloads, or additional jobs to meet household demands, potentially increasing exposure to occupational risk factors. Before data collection, the questionnaire underwent expert review by occupational health and epidemiology specialists to assess the relevance and clarity of the study items and enhance content validity. The instrument was subsequently pilot tested among 20 automobile repair artisans outside the study area to assess face validity and comprehensibility. Based on feedback from the pilot test, minor modifications were made before the commencement of the main survey. The internal consistency reliability of the questionnaire was assessed using Cronbach’s alpha, which yielded a coefficient of 0.76, indicating acceptable reliability. Throughout the data collection period, completed questionnaires were reviewed daily for completeness, consistency, and quality assurance. The collected data was exported to Microsoft Excel for cleaning and coding before being imported into Stata version 17 for statistical analysis. Variables with high rates of missing data were excluded from the analysis, while those with minimal missing data were addressed using appropriate statistical methods.

### Statistical analysis

The data were analyzed using the STATA statistical software version 17.0 [16]. Descriptive statistics such as frequencies, percentages, means, and standard deviations were used to describe the characteristics of the participants and the work-related factors, such as age, sex, job title, including auto mechanic, welder, sprayer, years of experience, and reported musculoskeletal symptoms in various body parts. Continuous variables were assessed for normality using the Shapiro–Wilk test. Variables that were not normally distributed (age, weight, and body mass index [BMI]) were summarized using the median and interquartile range (IQR), whereas normally distributed variables (height) were summarized using the mean and standard deviation (SD). Pearson’s chi-square test or Fisher’s exact test, where appropriate, was used to examine associations between categorical variables. Binary logistic regression analysis was performed to examine the association between the independent variables and the dependent variable. The dependent variable was the occurrence of self-reported work-related musculoskeletal disorders (WMSDs), defined as reporting pain, ache, discomfort, or numbness experienced in at least one anatomical body region during the previous 12 months that participants perceived to be related to their automobile repair work which was assessed using the Standardized Nordic Musculoskeletal Questionnaire (NMQ) that identifies musculoskeletal symptoms rather than clinically diagnosed disorders. Consequently, the dependent variable reflects the occurrence of self-reported work-related musculoskeletal symptoms and not physician-confirmed musculoskeletal disease. The dependent variable was coded as a binary variable (Yes = 1, No = 0). Body Mass Index (BMI) was calculated as body weight in kilograms divided by the square of height in meters (kg/m²). BMI was categorized according to the World Health Organization (WHO) [17]. classification for adults as follows: underweight (< 18.5 kg/m²), normal weight (18.5–24.9 kg/m²), overweight (25.0–29.9 kg/m²), and obesity (≥ 30.0 kg/m²). These categories were used to describe the nutritional status of the study participants and to assess their association with work-related musculoskeletal disorders. A graphical anatomical body map was used to illustrate the distribution of self-reported musculoskeletal symptoms across different body regions among automobile repair artisans. Variables with p-values less than 0.05 in the bivariate analysis were included in the logistic regression model to account for potential confounding. The adjusted odds ratios (aORs) with 95% confidence intervals were used to report the results, and the adequacy of the fitted logistic regression model was assessed using Pearson’s goodness-of-fit test. Statistical significance was set at *p* < 0.05. In this study, health burden was operationalized as Disability-Adjusted Life Years (DALYs). Because no mortality attributable to WMSDs was observed among the participants, the disability-adjusted life years (DALYs) were estimated using only years lived with disability (YLD). YLDs were estimated using the duration of symptoms and their respective disability weights. Mean values and standard deviations were calculated for the total DALYs and for each occupation. Total DALYs lost were estimated by multiplying the mean value of DALYs by the number of participants in each occupation category. Scores of Quality of Life (QoL) were described using mean ± SD and compared between occupations.

### Ethical consideration

Ethical clearance (CHRPE/AP/1143/25) was obtained from the Committee on Human Research, Ethics and Publications (CHRPE) of Kwame Nkrumah University of Science and Technology (KNUST). Written Informed consent was obtained from all participants before data collection. Participation was entirely voluntary, and respondents were informed of their right to withdraw at any time or to skip any questions without any negative consequences. All procedures were conducted in accordance with relevant ethical guidelines and regulations. Confidentiality and anonymity were ensured, as no identifying information was collected. Data were handled securely and used solely for academic purposes, with findings reported in aggregate form to prevent identification of individual participants.

## Results

### Socio-demographic characteristics of automobile repair artisans

The study involved 271 participants, predominantly male (95.9%), with a median age of 33 years. Most respondents were young adults aged 18–29 (40.6%). The average height was 1.66 m, median weight 67 kg, and median BMI 25.1 kg/m², with nearly half classified as overweight or obese (50.6%). Mechanics formed the largest occupational group (39.9%), followed by welders (27.3%). Almost half were married/divorced (47.2%), while educational attainment was concentrated at the JHS and SHS levels (28.4% each). Work experience varied, though most respondents had ≤ 20 years, and long working hours were common, with 72.4% reporting ≥ 9 h daily. Income distribution was polarized, with 36.5% earning ≤ 1000 GHS and 34.7% earning ≥ 2001 GHS (Table 1).

**Table 1:** Socio-demographic characteristics of automobile repair artisans

Variable	Frequency (*n* = 271)	Percentage (%)
Gender		
Female	11	4.1
Male	260	95.9
Age group in years	33(24–44)^a^	
18–29	110	40.6
30–39	82	30.3
40–49	44	16.2
50+	35	12.9
Height (meters)	1.66(± 0.11)^b^	
≤1.50	39	14.4
1.51–1.66	80	29.5
1.67–1.81	141	52.0
≥1.82	11	4.1
Weight (kg)	67(63–74)^a^	
≤59	30	11.1
60–71	152	56.1
72–82	74	27.3
≥83	15	5.5
Body mass index (BMI)	25.1(22.5–27.7)^a^	
Underweight (< 18.5 kg/m2)	14	5.2
Normal weight (18.5–24.9 kg/m2)	120	44.3
Overweight (25.0–29.9 kg/m2)	114	42.1
Obese (≥ 30.0 kg/ m2)	23	8.5
Type of work done		
Electrician	51	18.8
Mechanic	108	39.9
Sprayer	38	14.0
Welder	74	27.3
Marital status		
Single	108	39.9
Cohabiting	35	12.9
Married/ Divorced	128	47.2
Religion		
Christianity	171	63.1
Islamic	83	30.6
Traditionalist	17	6.3
Number of children		
0	102	37.6
1–2	72	26.6
3–4	66	24.4
5+	31	11.4
Education level		
No formal	41	15.1
Primary	51	18.8
JHS	77	28.4
SHS	77	28.4
Tertiary	25	9.2
Years of experience in years		
≤5	81	29.9
6–10	65	24.0
11–20	78	28.8
21+	47	17.3
Number of work hours per day		
≤6	9	3.3
7–8	66	24.4
9–10	133	49.1
≥11	63	23.3
Income level in GHS		
≤1000	99	36.5
1001–1500	40	14.8
1501–2000	38	14.0
>2001	94	34.7

*JHS*= Junior High School, *SHS*= Senior High School, *GHS*=Ghana Cedis

a=Median (Interquartile Range), b=Mean (Standard deviation)

### Prevalence and anatomical distribution of work-related musculoskeletal disorders among automobile repair artisans

**Fig. 1:**
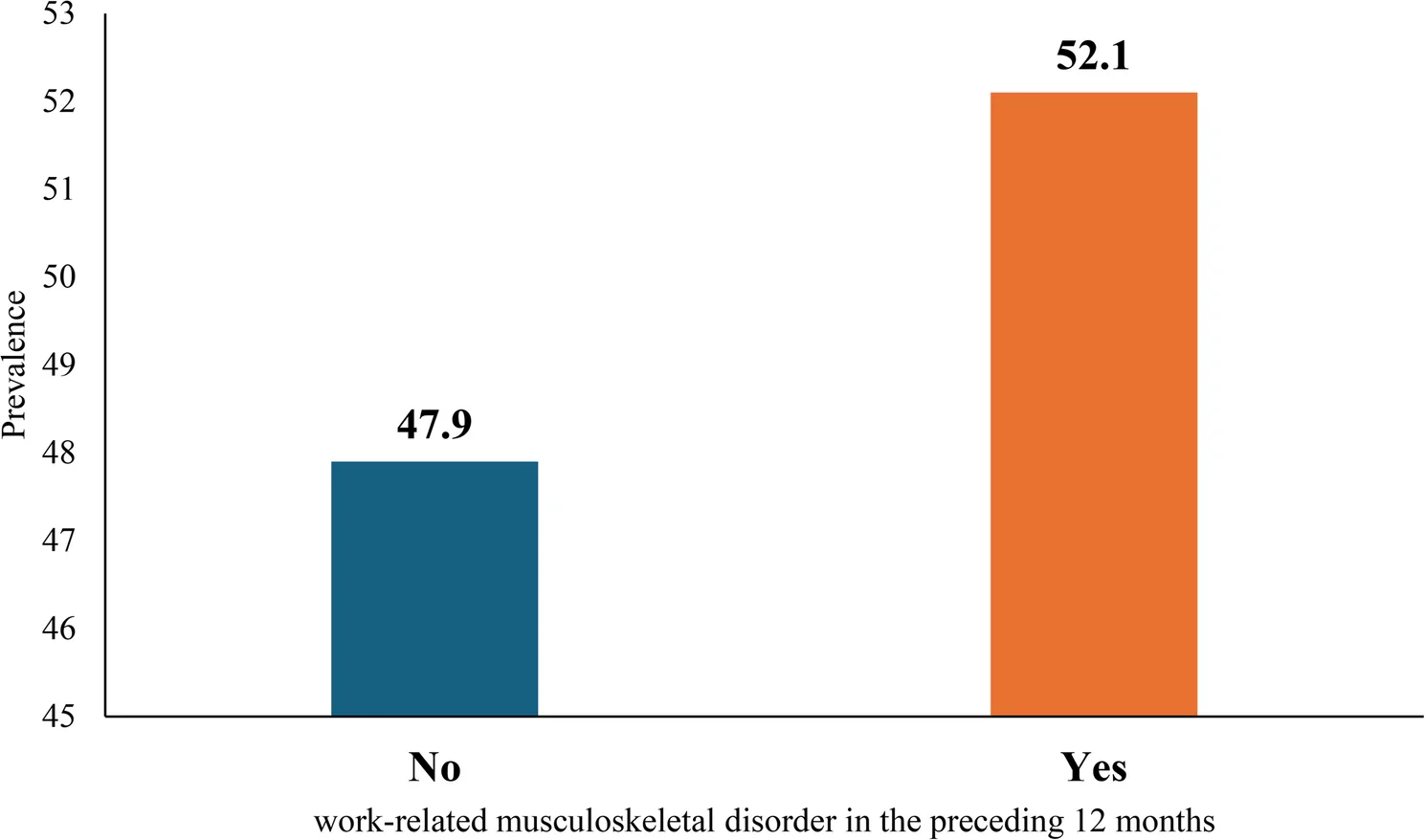
Twelve-month prevalence of musculoskeletal symptoms among automobile repair artisans

**Fig. 2:**
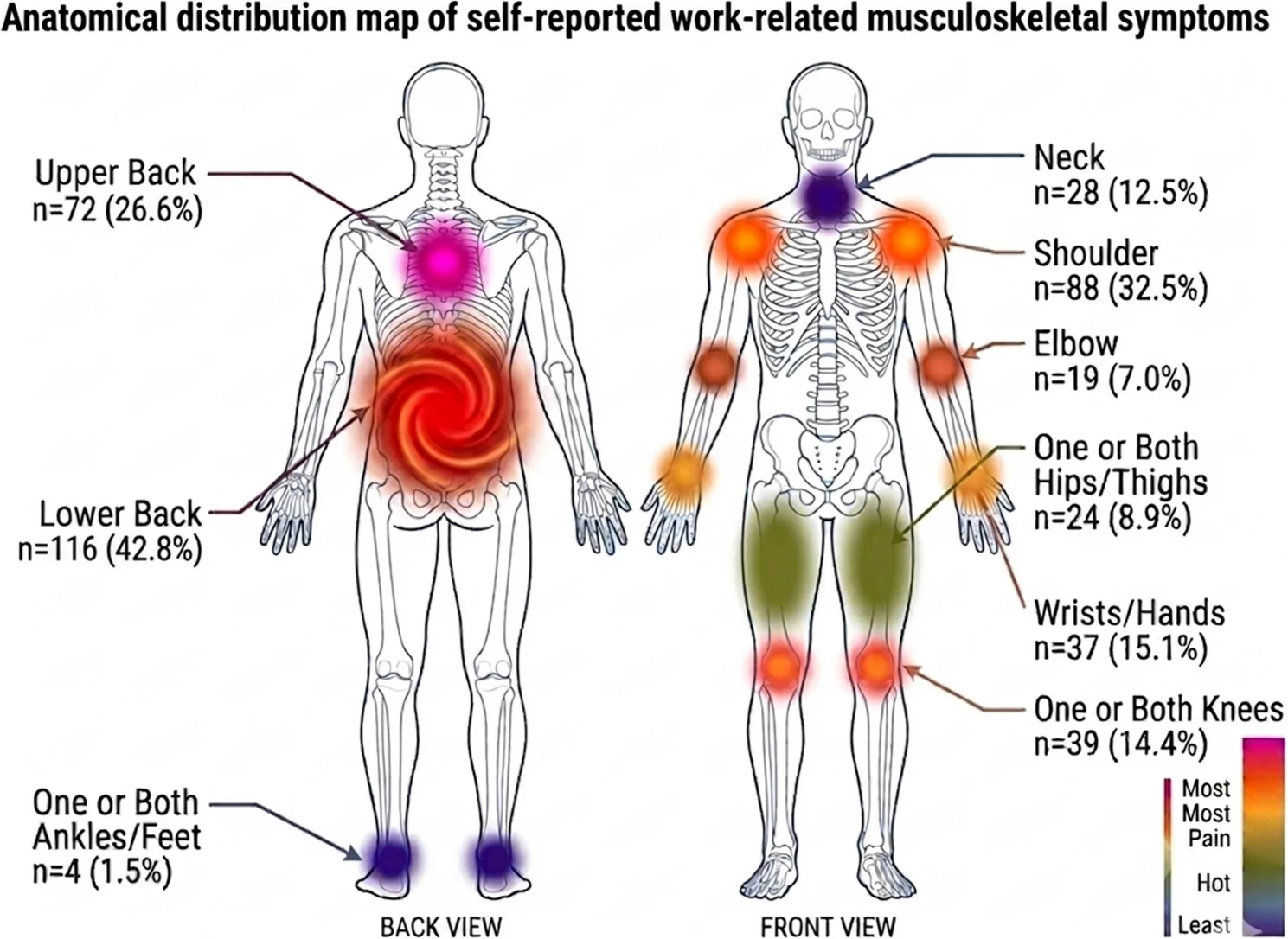
Anatomical distribution map of self-reported work-related musculoskeletal symptoms among automobile repair artisans

Overall, 52.1% of auto mobile repair artisans reported experiencing at least one work-related musculoskeletal disorder in the preceding 12 months (Fig. 1). The most affected area was the lower back (42.8%), followed by the shoulders (32.5%) and upper back (26.6%). Other reported areas included the wrists/hands (15.1%), knees (14.4%), neck (12.5%), and elbow (7.0%), with the least affected being the ankles/feet (1.5%). The anatomical distribution and severity of WMSDs (Fig. 2).

### Occupational-related factors of work-related musculoskeletal disorders (WMSDs) among automobile repair artisans

Table 2 presents multiple occupational risk factors associated with musculoskeletal disorders among artisans. Ergonomic risks were highly prevalent, with 94.8% reporting frequent bending or twisting of the back, 77.1% working with hands above shoulder level, and 90.0% squatting or kneeling for prolonged periods. Physical workload factors were also common: 41.0% lifted loads heavier than 20 kg without assistance, 50.5% pushed or pulled heavy vehicle parts, 73.4% performed repetitive hand/wrist tasks, and 66.8% used vibrating tools or machines. Regarding work organization, 83.4% usually worked in prolonged standing positions and 82.7% lacked mechanical aid to reduce manual effort. Nearly 40% worked more than 4 consecutive hours without rest, and 42.8% reported taking no breaks during a typical workday. Psychosocial stressors were prominent, with 90.8% feeling pressure to complete tasks quickly and 84.5% reporting stress or anxiety related to work. Despite these challenges, most participants indicated receiving support from colleagues (92.6%) and expressed overall job satisfaction (93.4%).

**Table 2:** Occupational-related factors of work-related musculoskeletal disorders (WMSDs) among automobile repair artisans

Variable		Frequency (*n* = 271)	Percentage (%)
Bend or twist your back while working			
No		14	5.2
Yes		257	94.8
Frequently work with your hands above shoulder level			
No		62	22.9
Yes		209	77.1
Squat or kneel for long hours during tasks (e.g. wheel or brake works)			
No		27	10.0
Yes		244	90.0
Lift load heavier than 20 kg without assistance			
No		160	59.0
Yes		111	41.0
Push or pull heavy vehicle parts (e.g. engines, gearboxes, tires)			
No		134	49.5
Yes		137	50.5
Perform repetitive hand/ wrist tasks (e.g. screwing, ratcheting)			
No		72	26.6
Yes		199	73.4
Use vibrating tools or machines			
No		90	33.2
Yes		181	66.8
Usually work in prolonged standing positions			
No	45		16.6
Yes	226		83.4
Lack mechanical aid to reduce manual effort			
No	47		17.3
Yes	224		82.7
Number of consecutive hours typically work without rest			
Less than 2 h	70		25.8
2–4 h	94		34.7
More than 4 h	107		39.5
Number of breaks usually take during a normal workday			
None	116		42.8
1–2	109		40.2
3 or more	46		17.0
Feel pressure to complete tasks quickly			
No		25	9.2
Yes		246	90.8
Feel stressed or anxious because of the work			
No		42	15.5
Yes		229	84.5
Receive enough support from colleagues when tasks are difficult			
No		20	7.4
Yes		251	92.6
Satisfied with the job overall			
No		18	6.6
Yes		253	

### Bivariate and multivariate logistic regression of socio-demographic related risk factors influencing the prevalence of work-related musculoskeletal disorders (WMSDs) automobile repair artisans

Table 3 shows the socio-demographic predictors of WMSDs. In the multivariate analysis, only height and income level remained significant predictors. Auto mechanics with height 1.67–1.81 m had higher adjusted odds of WMSDs (aOR = 3.42, 95% CI: 1.43–8.15, *p* = 0.006) compared with those ≤ 1.50 m. Similarly, those earning 1501–2000 GHS had increased odds (aOR = 4.61, 95% CI: 1.68– 12.7, *p* = 0.003) compared with those earning ≤ 1000 GHS. Other factors lost statistical significance after adjustment.

**Table 3:** Bivariate and multivariate logistic regression of socio-demographic related risk factors influencing the prevalence of work-related musculoskeletal disorders (WMSDs) among automobile repair artisans

Variable	Prevalence of WMSDs		Chi2(*p*-value)/ fishers exact	cOR (CI 95%), *p*-value	aOR (CI 95%), *p*-value
	No	Yes			
Gender			0.03(0.865)		
Female	5(45.5)	6(54.6)		Ref	
Male	125(48.1)	135(51.9)		0.9(0.27–3.02), 0.865	
Age group in years			25.1(**< 0.001**)		
18–29	68(61.8)	42(38.2)		0.21(0.09–0.50), **< 0.001**	1.10(0.19–6.38), 0.917
30–39	42(51.2)	40(48.8)		0.33(0.14–0.79), **0.013**	0.61(0.13–3.01), 0.546
40–49	11(25.0)	33(75.0)		1.04(0.38–2.88), 0.942	1.59(0.42–5.99), 0.491
50+	9(25.7)	26(74.3)		Ref	Ref
Height (meters)			**0.049**		
≤ 1.50	26(66.7)	13(33.3)		Ref	Ref
1.51–1.66	40(50.0)	40(50.0)		2.0(0.90–4.43), 0.088	2.34(0.95–5.76), 0.065
1.67–1.81	60(42.6)	81(57.5)		2.7(1.28–5.69), **0.009**	3.42(1.43–8.15), **0.006**
≥ 1.82	4(36.4)	7(63.6)		3.5(0.87–14.2), 0.079	1.59(0.42–5.99), 0.491
Weight (kg)			3.04(0.385)		
≤ 59	15(50.0)	15(50.0)		Ref	
60–71	79(52.0)	73(48.0)		0.92(0.42–2.02), 0.843	
72–82	30(40.5)	44(59.5)		1.47(0.63–3.44), 0.379	
≥ 83	6(40.0)	9(60.0)		1.5(0.43–5.27), 0.527	
Body mass index (BMI) in kg/m2			0.268		
Underweight (< 18.5)	4(28.6)	10(71.4)		Ref	
Normal weight (18.5–24.9)	54(45.0)	66(55.0)		0.49(0.15–1.65), 0.248	
Overweight (25.0–29.9)	61(53.5)	53(46.5)		0.35(0.10–1.17), 0.089	
Obese (≥ 30.0)	11(47.8)	12(52.2)		0.44(0.11–1.80), 0.252	
Type of work done			3.59(0.309)		
Electrician	29(56.9)	22(43.1)		Ref	
Mechanic	54(50.0)	54(50.0)		1.32(0.67–2.58), 0.419	
Sprayer	17(44.7)	21(55.3)		1.63(0.70–3.80), 0.259	
Welder	30(40.5)	44(59.5)		1.93(0.94–3.98), 0.074	
Marital status			28.2(**< 0.001**)		
Single	73(67.6)	35(32.4)		Ref	Ref
Cohabiting	14(40.0)	21(60.0)		3.13(1.42–6.87), **0.005**	1.95(0.73–5.22), 0.187
Married/ Divorced	43(33.6)	85(66.4)		4.12(2.39–7.11), **< 0.001**	2.46(0.98–6.16), **0.054**
Religion			4.26(0.119)		
Christianity	89(52.1)	82(48.0)		Ref	
Islamic	32(38.6)	51(61.5)		1.73(1.01–2.95), 0.054	
Traditionalist	9(52.9)	8(47.1)		0.97(0.36–2.62), 0.944	
Number of children			28.2(**< 0.001**)		
0	69(67.7)	33(32.4)		Ref	Ref
1–2	28(38.9)	44(61.1)		3.29(1.75–6.17), **< 0.001**	1.57(0.64–3.88), 0.327
3–4	26(39.4)	40(60.6)		3.22(1.69–6.13), **< 0.001**	1.38(0.47-4.0), 0.558
5+	7(22.6)	24(77.4)		7.17(2.80–18.3), **< 0.001**	2.02(0.47–8.67), 0.346
Education level			4.61(0.330)		
No formal	19(46.3)	22(53.7)		Ref	
Primary	23(45.1)	28(54.9)		1.05(0.46–2.40), 0.905	
JHS	31(40.3)	46(59.7)		1.28(0.60–2.75), 0.525	
SHS	43(55.8)	34(44.2)		0.68(0.32–1.46), 0.326	
Tertiary	14(56.0)	11(44.0)		0.68(0.25–1.85), 0.447	
Years of experience in years			22.5(**< 0.001**)		
≤ 5	53(65.4)	28(34.6)		Ref	Ref
6–10	33(50.8)	32(49.2)		1.84(0.94–3.58), 0.075	1.15(0.48–2.73), 0.755
11–20	33(42.3)	45(57.7)		2.58(1.36–4.90), **0.004**	0.70(0.24–2.07), 0.521
21+	11(23.4)	36(76.6)		6.20(2.74–14.01), **< 0.001**	1.21(0.24–6.08), 0.817
Number of work hours per day			**0.013**		
≤ 6	6(66.7)	3(33.3)		Ref	
7–8	42(63.6)	24(36.4)		1.14(0.26–4.99), 0.859	
9–10	55(41.4)	78(58.7)		2.84(0.68–11.8), 0.153	
≥ 11	27(42.9)	36(57.1)		2.67(0.61–11.6), 0.192	
Income level in GHS			26.8(**< 0.001**)		
≤ 1000	66(66.7)	33(33.3)		Ref	Ref
1001–1500	21(52.5)	19(47.5)		1.81(0.86–3.82), 0.120	1.70(0.66–4.35), 0.272
1501–2000	12(31.6)	26(68.4)		4.33(1.94–9.66), **< 0.001**	4.61(1.68–12.7), **0.003**
≥ 2001	31(33.0)	63(67.0)		4.07(2.23–7.40), **< 0.001**	2.11(0.87–5.13), 0.100

*aOR* Adjusted Odds Ratio, *CI* Confidence Interval, *Ref* Reference category

*p*-value: Statistical significance level; Bolded values (*p* < 0.05) indicate statistically significant associations: Pearson test for goodness of fit (*p* = 0.3908); Number of Covariate patterns (162); Pearson Chi2 (148.1)

### Bivariate and Multivariate Logistic Regression of Occupational related risk factors influencing the prevalence of work-related musculoskeletal disorders (WMSDs) among automobile repair artisans

Table 4 examined the association between work-related risk factors and the prevalence of WMSDs among auto mechanics. In the multivariate model, two factors remained significant. Auto mechanics who reported using vibrating tools or machines had lower odds of WMSDs (aOR = 0.56, 95% CI: 0.31–0.99, *p* = 0.049), while job satisfaction was protective, with dissatisfied artisans significantly less likely to report WMSDs compared with those satisfied (aOR = 0.19, 95% CI: 0.05–0.66, *p* = 0.009).

**Table 4:** Bivariate and Multivariate Logistic Regression of Occupational related risk factors influencing the prevalence of work-related musculoskeletal disorders (WMSDs) among automobile repair artisans

Variable	Prevalence of WMSDs		Chi2(*p*-value)/ fishers exact	cOR (CI 95%), *p*-value	aOR (CI 95%), *p*-value
	No	Yes			
Often bend or twist your back while working			0.50(0.481)		
No	8(57.1)	6(42.9)		Ref	
Yes	122(47.5)	135(52.5)		1.48(0.50–4.37), 0.483	
Frequently work with your hands above shoulder level			0.43(0.513)		
No	32(51.6)	30(48.4)		Ref	
Yes	98(46.9)	111(53.1)		1.21(0.69–2.13), 0.514	
Squat or kneel for long hours during tasks such as wheel or brake works			0.63(0.428)		
No	11(40.7)	16(59.3)		Ref	
Yes	119(48.8)	125(51.2)		0.72(0.32–1.62), 0.430	
Lift load heavier than 20 kg without assistance			0.31(0.578)		
No	79(49.4)	81(50.6)		Ref	
Yes	51(46.0)	60(54.1)		1.15(0.71–1.86), 0.579	
Push or pull heavy vehicle parts such as engines, gearboxes, tires			1.65(0.199)		
No	59(44.0)	75(56.0)		Ref	
Yes	71(51.8)	66(48.2)		0.73(0.45–1.18), 0.200	
Perform repetitive hand/ wrist tasks such as screwing, ratcheting			0.16(0.687)		
No	36(50.0)	36(50.0)		Ref	
Yes	94(47.2)	105(52.8)		1.12(0.65–1.92), 0.688	
Use vibrating tools or machines			2.54(0.111)		
No	37(41.1)	53(58.9)		Ref	Ref
Yes	93(51.4)	88(48.6)		0.66(0.40–1.10), 0.112	0.56(0.31–0.99), **0.049**
Usually work in prolonged standing positions			0.037(0.848)		
No	21(46.7)	24(53.3)		Ref	
Yes	109(48.2)	117(51.8)		0.94(0.50–1.78), 0.848	
Lack mechanical aid to reduce manual effort			3.17(0.075)		
No	17(36.2)	30(63.8)		Ref	
Yes	113(50.5)	111(49.6)		0.56(0.29–1.07), 0.077	

*aOR* Adjusted Odds Ratio, *CI* Confidence Interval, *Ref* Reference category

*p*-value: Statistical significance level; Bolded values (*p* < 0.05) indicate statistically significant associations: Pearson test for goodness of fit (*p* = 0.1119); Number of Covariate patterns (196); Pearson Chi2 (201.2)

### Disability adjusted life years (DALYs) lost among automobile repair artisans

The overall mean DALY was 0.230 (SD = 0.238), corresponding to a total of 62.21 DALYs lost in the study population. Welders recorded the highest mean DALY (0.303) and total DALYs lost (22.39), followed closely by mechanics (mean = 0.256; total = 24.38). Electricians and sprayers had comparatively lower mean DALYs of 0.181 and 0.163, respectively, with total DALYs loss of 9.24 and 6.21. Despite these differences, mean QoL scores were relatively similar across occupational groups, ranging from 3.11 among mechanics to 3.21 among electricians, with an overall mean QoL of 3.16 (SD = 0.36) (Table 5).

**Table 5:** Disability adjusted life years (DALYs) lost due to work-related musculoskeletal disorders by occupation (*n* = 271)

Occupation	*N*	Mean DALY	Median	SD	Total DALYs	Mean QoL	Median QoL	SD QoL
Electrician	51	0.181	0.080	0.200	9.24	3.21	3.25	0.23
Mechanic	108	0.256	0.080	0.236	24.38	3.11	3.13	0.40
Sprayer	38	0.163	0.060	0.191	6.21	3.15	3.25	0.34
Welder	74	0.303	0.188	0.269	22.39	3.19	3.25	0.37
Total	271	0.230	0.080	0.238	62.21	3.16	3.25	0.36

Disability-adjusted life years (*DALYs*) were estimated as years lived with disability (*YLD*) only, since no mortality (*YLL*) was observed in this cross-sectional sample of artisans and Quality of Life (*QoL*); Total DALYs lost=Mean×N

## Discussion

This research makes it clear that there is a great burden of work-related musculoskeletal disorders (WMSDs) among the informal auto repair artisans in the Sunyani Municipality of Ghana. More than half of the respondents (52.1%) reported that they had experienced WMSDs in the past 12 months. This is similar to what has been found in previous studies among the informal auto mechanics in Ghana and other low- and middle-income countries, where the reported prevalence ranges from 50% to 75% [9, 10, 15]. The most affected parts of the body were the lower back, shoulders, and upper back. This is similar to the physical demands of the job of informal auto repair artisans. Activities such as frequent bending and twisting, standing for long periods, overhead work, and manual handling of heavy vehicle parts put excessive biomechanical loads on the spine and upper limbs, making them susceptible to musculoskeletal injuries [3, 11]. Comparable distributions have also been found among auto mechanics and other related occupations in Africa and Asia, where spinal and shoulder problems are the most common [9]. Notably, more than 80% of artisans with WMSDs experienced symptoms in the past seven days, indicating a continuous exposure to dangerous work practices and limited opportunities for recovery.

In multivariable analysis, several socio-demographic and occupational variables were found to be independently associated with WMSDs. Artisans with heights of 1.67 to 1.81 m had significantly higher odds of WMSDs, possibly due to a mismatch between the workers’ and non-adjustable vehicle parts, workbenches, or ground-level activities that are often seen in informal workshops [18].Comparable relationships between anthropometry and musculoskeletal risk have also been found in ergonomic studies involving informal and artisanal workers, where workstations are not designed to fit the body dimensions of the workers [11].

Income level was also found to be a predictor, with artisans in the GHS 1501–2000 income level having higher odds of WMSDs. This could be due to increased working hours, workload, or the need to earn a living through physically demanding jobs, which is common among informal sector workers in sub-Saharan Africa [5, 11]. High levels of exposure to ergonomic risk factors such as bending or twisting, standing for long periods, repetitive hand or wrist movements, and the use of vibrating tools were common, which was expected given the nature of similar occupations [15, 19]. The use of vibrating tools had an adjusted odds ratio of less than one, indicating a protective effect. This result should be treated with caution. Likely, the result does not reflect a genuine protective effect but rather task specialization, intermittent exposure, or the substitution of manual force with powered tools, which reduces overall physical effort. There is extensive evidence of the adverse effects of high-intensity vibration exposure on the upper limbs and spine [3]. Similarly, job dissatisfaction was associated with lower odds of reported WMSDs. Notwithstanding, this could be a reflection of behavioral reactions such as absenteeism, task avoidance, or reduced exposure among disgruntled employees. In informal occupational environments, psychosocial issues could affect both exposure and symptom reporting. Previous studies have shown that psychosocial stressors mediate the relationship between physical workload and musculoskeletal outcomes, but these relationships are not necessarily linear [18–20]. These findings emphasize the need for careful interpretation of psychosocial issues in informal occupational environments. In addition to prevalence and risk factors, this study also quantified the disability burden of WMSDs in terms of disability-adjusted life years (DALYs). A total of 62.21 DALYs were lost in the study population, with the highest burden in mechanics and welders. This is due to the increased physical demands of these occupations and the associated prolonged exposure to ergonomic risks. The results are consistent with international evidence that musculoskeletal disorders have identified as a major contributor to years lived with disability, especially in manual and informal workers [2]. The documented productivity losses, work limitations, and diminished quality of life further emphasize the socioeconomic impact of WMSDs on artisans and their families. In conclusion, the results of this study clearly show that WMSDs among informal auto repair artisans are mainly caused by modifiable ergonomic and organizational factors in poorly regulated work environments. By taking a multi-trade approach in one informal automobile workshop cluster, the current study closes an important gap in the literature, which has mainly concentrated on single trade independently. Ergonomic work modifications, improved work organization, and the inclusion of informal workers into occupational health policies are crucial to diminish preventable disability and enhance long-term health and productivity in this at-risk workforce.

### Strength and limitation

A major strength of this study is the inclusion of multiple auto-related trades within the same informal workshop cluster, providing a broader assessment of WMSDs than studies focusing on a single occupational group. The use of a standardized and validated instrument (Nordic Musculoskeletal Questionnaire) increases the validity of the results. In addition, the use of disability-adjusted life years (DALYs) provides new data on the general health and productivity effects of WMSDs in informal auto repair artisans. However, there are a number of limitations that should be considered. First, the cross-sectional design of the study limits the ability to establish the causal relationships between the identified risk factors and WMSDs, and the results should be interpreted in terms of association rather than causation. Second, the study was based on self-reported information regarding musculoskeletal symptoms and work exposures, which could be prone to recall bias, especially regarding symptoms reported over the period of 12 months. Third, because WMSDs were assessed using self-reported symptoms rather than clinical examination or diagnostic confirmation, some non-occupational musculoskeletal conditions may not have been completely distinguishable from occupationally related symptoms, potentially resulting in misclassification bias. Although individuals with a self-reported history of musculoskeletal conditions unrelated to occupational exposure were excluded from participation, residual misclassification cannot be entirely ruled out. There is also the possibility of underreporting, as pain and discomfort are often normalized among informal workers. Finally, because the study was conducted within the largest automobile repair cluster in the municipality, artisans may have experienced higher workloads and occupational exposures than workers in smaller repair settings. Consequently, the findings should be generalized to other informal automobile repair populations with caution.

## Conclusions

The current study established a high prevalence of work-related musculoskeletal disorders (WMSDs) among the informal auto repair artisans in the Sunyani Municipality in Ghana, where more than half of the workers experienced WMSDs in the last 12 months. The body parts most often affected were the lower back, shoulders, and upper back, which may be attributed to the physical demands of the work. Height and income levels were established as predictors of WMSDs, while the workers were exposed to various risk factors. The estimated disability-adjusted life years established a high burden of disability, especially among the mechanics and welders. We therefore recommend the need to address the situation by implementing various low-cost ergonomic interventions, work organization, basic occupational health and safety education, and the gradual inclusion of the artisans in the national occupational health and safety policies.

We are grateful to the Sunyani Municipal Assembly and the executive leadership of the Sunyani Magazine for their administrative support and for facilitating access to the study site. We express our profound gratitude to the informal automobile artisans of Sunyani Magazine for their time and willingness to participate in this study. We also acknowledge the academic guidance provided by the faculty of the Kwame Nkrumah University of Science and Technology (KNUST) throughout the research process.

## Supplementary Information

Supplementary Material 1.

## Data Availability

The data supporting the findings of this study are available on request from the corresponding authors.

## Abbreviations

WMSDs: Work-Related Musculoskeletal Disorders
MSDs: Musculoskeletal Disorders
DALYs: Disability-Adjusted Life Years
YLL: Years of Life Lost
QoL: Quality of Life
YLD: Years Lived with Disability
NMQ: Nordic Musculoskeletal Questionnaire
