# Supplementary material for: Prevalence, health burden, and occupational risk factors of work-related musculoskeletal disorders among informal automobile repair artisans in Sunyani Municipality, Ghana: a cross-sectional study
Source: BMC Public Health. 2026 Jul 8;26:2724. doi: 10.1186/s12889-026-28478-8 (PMC13628820; doi:10.1186/s12889-026-28478-8)
Supplement: Supplementary file 1 [file 12889_2026_28478_MOESM1_ESM.pdf]

KWAME NKRUMAH UNIVERSITY OF SCIENCE AND TECHNOLOGY, (K.N.U.S.T)

COLLEGE OF HEALTH SCIENCES

DEPARTMENT OF OCCUPATIONAL AND ENVIRONMENTAL HEALTH AND SAFETY

Dear Respondent,

I am a final year MSc student carrying out research on the topic "Musculoskeletal Disorders among Automobile Mechanics at the Sunyani Magazine". This is purely for academic purposes. I will be very grateful, therefore, if you would provide answers to the following questions. Please be assured that the answers provided will be treated confidentially. Your identity is not required.

SECTION I: Socio-Demographic Characteristics of Respondents

INSTRUCTION: *Please tick (√) to indicate your choice or response to a question and fill in the appropriate answer in spaces where options are not available.*

1. Gender [ ] Male [ ] Female
2. Age [ ]
3. What is your height (meters)? [ ]
4. What is your weight (kg) [ ]
5. What is your estimated body mass index (BMI)? [ ]
6. Relationship status [ ] single [ ]married [ ] divorced [ ] widowed [ ] cohabitation
7. Religion [ ] None [ ] Christianity [ ] Islamic [ ] Traditionalist
8. Number of children [ ]

9. Education Qualification [ ] None [ ] basic [ ] JHS [ ] SHS
10. Years of experience as a mechanic [ ]
11. Type of work done, tick all that apply

NO	Category of work	Please appropriately tick (√)
1.	MECHANIC	☐
2.	WELDER/ STRAIGHTER	☐
3.	SPRAYER	☐
4.	ELECTRICIAN	☐
5.	Others…………	

12. The number of work hours per day [ ]
13. Income level [ ]

Musculoskeletal Discomfort Form (Based on the Nordic Questionnaire (Kourinka et al. 1987)) **Employee ID:**________________

Job/Position: ______________________ Gender: M F Age: _______ Height: ____ ft.____ in. Weight: ____________
How long have you been doing this job? ___years ___ months How many hours do you work each week? _____

How to answer the questionnaire:

***Picture*:** In this picture you can see the approximate position of the parts of the body referred to in the table. Limits are not sharply defined, and certain parts overlap. You should decide for yourself in which part you have or have had your trouble (if any).

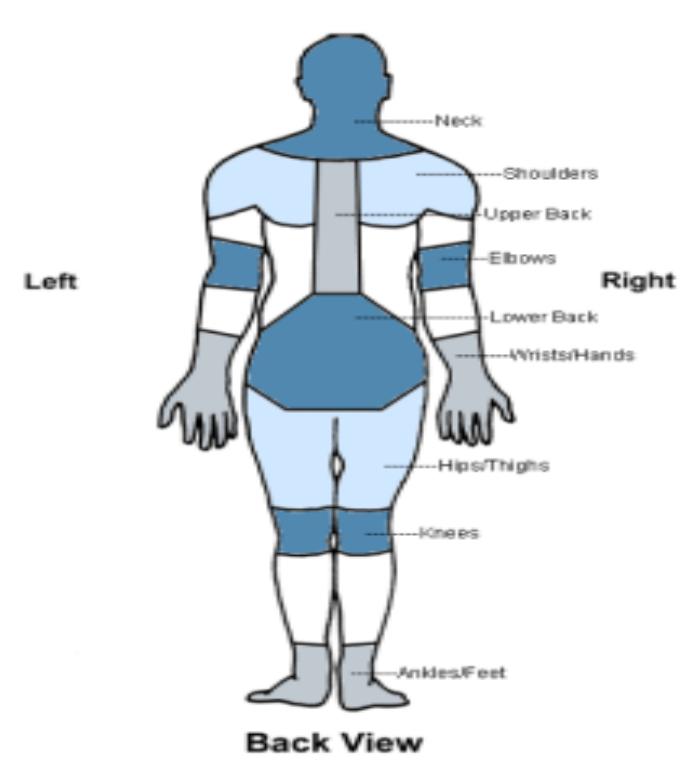

***Table*:** Please answer by putting an "X" in the appropriate box - one "X" for each question. You may be in doubt as to how to answer, but please do your best anyway. Note that column 1 of the questionnaire is to be answered even if you have never had trouble in any part of your body; columns 2 and 3 are to be answered if you answered yes in column 1.

To be answered by everyone		To be answered by those who have had trouble			
Have you at any time during **the last 12 months** had trouble (ache, pain, discomfort, numbness) in:		Have you at any time during the **last 12 months been prevented from doing your normal work** (at home or away from home) because of the trouble?		Have you had trouble at any time during **the last 7 days?**	
**Neck**<br>☐ No	☐ Yes	☐ No	☐ Yes	☐ No	☐ Yes
**Shoulders**<br>☐ No	☐ Yes, right shoulder<br>☐ Yes, left shoulder<br>☐ Yes, both shoulders	☐ No	☐ Yes	☐ No	☐ Yes
**Elbows**<br>☐ No	☐ Yes, right elbow<br>☐ Yes, left elbow<br>☐ Yes, both elbows	☐ No	☐ Yes	☐ No	☐ Yes
**Wrists/Hands**<br>☐ No	☐ Yes, right wrist/hand<br>☐ Yes, left wrist/hand<br>☐ Yes, both wrists/hands	☐ No	☐ Yes	☐ No	☐ Yes
**Upper Back**<br>☐ No	☐ Yes	☐ No	☐ Yes	☐ No	☐ Yes
**Lower Back (small of back)**<br>☐ No	☐ Yes	☐ No	☐ Yes	☐ No	☐ Yes
**One or Both Hips/Thighs**<br>☐ No	☐ Yes	☐ No	☐ Yes	☐ No	☐ Yes
**One or Both Knees**<br>☐ No	☐ Yes	☐ No	☐ Yes	☐ No	☐ Yes
**One or Both Ankles/Feet**<br>☐ No	☐ Yes	☐ No	☐ Yes	☐ No	☐ Yes

Section 2: Prevalence and symptoms of Work- Related Musculoskeletal Disorders on body parts

2.2 what was the severity of pain/discomfort experienced?

[ ] mild [ ] moderate [ ] severe

2.3 how long do these pain and/ discomfort typically last after experiencing them?

[ ] less than an hour [ ]few hours [ ] half a day [ ]full day [ ] longer than a day

2.4 how many years have you been living with work-related pain or discomfort? [ ]

2.5 Are there specific activities or tasks that seem to trigger or worsen your pain or discomfort?

[ ]Yes [ ] No

Section 3: Work-Related Risk factors

Ergonomic risk factors

1.1 do you often bend or twist your back while working?

[ ] Yes [ ] No

1.2 do you frequently work with your hands above shoulder level?

[ ] Yes [ ]No

1.3 do you spend long hours lying on your back or stomach under vehicles?

[ ] Yes [ ] No

1.4 do you squat or kneel for long hours during tasks (e.g. wheel or brake works)?

[ ] Yes [ ] No

Physical factors

1.5 do you lift load heavier than 20kg without assistance?

[ ] Yes [ ] No

1.6 do you push or pull heavy vehicle parts (e.g. engines, gearboxes, tires)?

[ ] Yes [ ] No

1.7 do you perform repetitive hand/ wrist tasks (e.g. screwing, ratcheting)

[ ] Yes [ ] No

1.8 do you use vibrating tools or machines?

[ ] Yes [ ]No

Work organization factors

1.9 do you usually work in prolonged standing positions?

[ ] Yes [ ] No

1.10 do you lack mechanical aid to reduce manual effort?

[ ] Yes [ ] No

1.11 How many consecutive hours do you typically work without rest?

[ ] less than 2 hrs. [ ] 2-4 hours [ ] more than 4 hours

1.12 how many breaks do you usually take during a normal workday?

[ ] None [ ] 1-2 [ ] 3 or more

Psychosocial factors

1.13 do you feel pressure to complete tasks quickly?

[ ] Yes [ ] No

1.14 do you feel stressed or anxious because of your work?

[ ] Yes [ ] No

1.15 do you receive enough support from colleagues when tasks are difficult?

[ ] Yes [ ] No

1.16 are you satisfied with your job overall?

[ ] Yes [ ] No

Section 4: Quality of life (physical health, psychological, social relationships, and environment)

4.1 Do you have enough energy for daily life?

[ ] Not at all [ ]A little [ ]moderately [ ]mostly [ ] completely

4.2 to what extent do pain prevents you from doing what you need to do ?

[ ]Not at all [ ] A little [ ]Moderately [ ]Mostly[ ]Completely

4.3 how often do you feel negative feelings(blue mood, despair, anxiety, depression) ?

[ ]Never [ ]Seldom [ ]Quite Often [ ]Very Often[ ]Always

4.4 How satisfied are you with your personal relationships and support?

[ ]Very Dissatisfied[ ]Dissatisfied[ ]Neutral[ ]Satisfied[ ]Very Satisfied

4.5 how safe do you feel in your daily life at work ?

[ ]Not Safe[ ]Slightly Safe[ ]Moderately Safe[ ]Very Safe

4.6 in the past 12 months, how many days did you loose due to musculoskeletal problems?

[ ]None [ ]1-7 Days[ ]8-30days [ ]More than 30 days

4.7 on a typical day with WMSD pain, how limited are you in performing normal activities ?

[ ]Not Limited [ ]Mildly Limited[ ]Severely Limited[ ]Completely Unable

4.8 Compared with a healthy person as your age, how would you rate your health today?

[ ]Perfect Health [ ]0.75 [ ]0.5 [ ]Death Equivalent

4.9 Duration of symptoms (for YLD)?

[ ]longer than a day[ ]less than 1 month [ ]1-3 months [ ]4-6 months

[ ] more than 6 months

Conclusion: Thank you for completing this questionnaire.
